# Novel Approach for Detecting Respiratory Syncytial Virus in Pediatric Patients Using Machine Learning Models Based on Patient-Reported Symptoms: Model Development and Validation Study

**DOI:** 10.2196/52412

**Published:** 2024-04-12

**Authors:** Shota Kawamoto, Yoshihiko Morikawa, Naohisa Yahagi

**Affiliations:** 1 Graduate School of Media and Governance Keio University Fujisawa Japan

**Keywords:** respiratory syncytial virus, machine learning, self-reported information, clinical decision support system, decision support, decision-making, artificial intelligence, model development, evaluation study, detection, respiratory, respiratory virus, virus, machine learning model, pediatric, Japan, detection model

## Abstract

**Background:**

Respiratory syncytial virus (RSV) affects children, causing serious infections, particularly in high-risk groups. Given the seasonality of RSV and the importance of rapid isolation of infected individuals, there is an urgent need for more efficient diagnostic methods to expedite this process.

**Objective:**

This study aimed to investigate the performance of a machine learning model that leverages the temporal diversity of symptom onset for detecting RSV infections and elucidate its discriminatory ability.

**Methods:**

The study was conducted in pediatric and emergency outpatient settings in Japan. We developed a detection model that remotely confirms RSV infection based on patient-reported symptom information obtained using a structured electronic template incorporating the differential points of skilled pediatricians. An extreme gradient boosting–based machine learning model was developed using the data of 4174 patients aged ≤24 months who underwent RSV rapid antigen testing. These patients visited either the pediatric or emergency department of Yokohama City Municipal Hospital between January 1, 2009, and December 31, 2015. The primary outcome was the diagnostic accuracy of the machine learning model for RSV infection, as determined by rapid antigen testing, measured using the area under the receiver operating characteristic curve. The clinical efficacy was evaluated by calculating the discriminative performance based on the number of days elapsed since the onset of the first symptom and exclusion rates based on thresholds of reasonable sensitivity and specificity.

**Results:**

Our model demonstrated an area under the receiver operating characteristic curve of 0.811 (95% CI 0.784-0.833) with good calibration and 0.746 (95% CI 0.694-0.794) for patients within 3 days of onset. It accurately captured the temporal evolution of symptoms; based on adjusted thresholds equivalent to those of a rapid antigen test, our model predicted that 6.9% (95% CI 5.4%-8.5%) of patients in the entire cohort would be positive and 68.7% (95% CI 65.4%-71.9%) would be negative. Our model could eliminate the need for additional testing in approximately three-quarters of all patients.

**Conclusions:**

Our model may facilitate the immediate detection of RSV infection in outpatient settings and, potentially, in home environments. This approach could streamline the diagnostic process, reduce discomfort caused by invasive tests in children, and allow rapid implementation of appropriate treatments and isolation at home. The findings underscore the potential of machine learning in augmenting clinical decision-making in the early detection of RSV infection.

## Introduction

Every winter, respiratory syncytial virus (RSV) causes acute lower respiratory tract infections in approximately 33.8 million children younger than 5 years worldwide [1]. Approximately all children are infected at least once, and half are infected twice or more by the age of 24 months [2]. Newborns and children with underlying medical conditions are particularly susceptible to severe infection [3-5]. Therefore, reducing the number of RSV-infected patients is paramount for reducing the number of associated deaths. Consequently, there is an urgent need to develop a quick and accurate detection system for RSV infection [6].

Laboratory testing of RSV, including rapid antigen testing and polymerase chain reaction tests, provides reasonably accurate infection-related information [7]. However, the collection of nasopharyngeal secretions, a necessary step for these tests, can cause discomfort in children. Furthermore, given that RSV test results rarely influence treatment decisions, these tests are not routinely conducted [8,9]. On a positive note, RSV infections are not serious in most cases, and there is no active treatment, indicating that most patients can be treated at home under the constant supervision of parents or caregivers while taking precautions [10,11]. Thus, remote identification of RSV infection allows patients to be cared for at home, preventing infection spread [12]. This approach could also ease the burden on health care workers during epidemics by providing remotely procured information. Nonetheless, a home-based detection method that matches the accuracy of a rapid antigen test has yet to be recognized.

The symptoms and signs of RSV infection may help establish remote detection strategies. Studies that focused on the detection of RSV infection based on symptoms either lacked discriminatory accuracy or highlighted difficulties because of the diverse clinical manifestations of RSV infection [13-18]. However, symptom onset of RSV infection may not appear as a cross-sectionally typical pattern at a specific time point but rather as a pattern that is diverse in characteristics, including the longitudinal aspects of symptoms. Particularly, the symptoms of RSV infection peak 4-5 days after infection and change with the increase or decrease in viral load [19,20]. Dyspnea and other lower respiratory symptoms, including wheezing, moaning, and tachypnea, which are typical symptoms of severe RSV infection, occur when infected ciliated bronchial epithelial cells drop into the lower respiratory tract, thereby delaying the manifestation of upper respiratory symptoms [21-23]. Contrarily, most studies linking RSV infection to overt symptoms used cross-sectional data based on symptoms at specific time points and did not consider the time course of symptom onset in the longitudinal profile of individual children.

Therefore, we propose that cross-sectional studies based on specific time points of signs and symptoms expressed with RSV infection appear to be unrelated to RSV infection, but this may not represent the unique disease trajectory of RSV infection in individual patients infected with RSV. Therefore, machine learning models based on symptom data structured to include longitudinal characteristics may enable highly accurate identification of viral infection. As the progression of symptoms can exhibit multiple patterns in each individual, considering aspects such as the size of patient bronchi and machine learning algorithms, which are already widely used to diagnose and classify diseases based on symptom characteristics, are equally suitable for identifying RSV infection [24-27]. Here, we sought to leverage the longitudinal diversity of symptoms using machine learning based on patient-reported information, aiming to confirm the presence of RSV infection remotely at home with sensitivity and specificity comparable to those of rapid antigen testing [28]. If this strategy is recognized, it will contribute to reducing the physical burden on children, saving medical costs, and preventing nosocomial infections. The purpose of this study is to develop and validate a machine learning–based RSV infection identification model using patient self-reported symptom information for outpatients. This study is also valuable as it is one of the few studies conducted in a cohort of outpatients with mild infections.

## Methods

### Overview

The results are presented according to the TRIPOD (Transparent Reporting of a Multivariable Prediction Model for Individual Prognosis or Diagnosis) statement [29].

### Ethical Considerations

The study was conducted according to the Declaration of Helsinki and Japan’s ethical guidelines. The institutional review board of Yokohama City Municipal Hospital approved the protocol (18-05-04), and we obtained informed consent from the patients’ parents in the form of an opt-out clause. In this study, analysis was conducted using data that had been anonymized to ensure the privacy and confidentiality of participants. No compensation was provided to the participants.

### Data Collection Setting

This observational retrospective cohort study involved patients aged ≤24 months who visited the pediatric or emergency department of Yokohama City Municipal Hospital between January 2009 and December 2015 and had RSV rapid antigen test results. According to the facility policy, all outpatients were required to fill an electronic template, and those who exhibited cold symptoms were subjected to RSV rapid antigen testing. These patients were extracted from a prospectively curated database and enrolled in this study. In this study, we used the immunochromatographic Quick-Navi RSV test (Denka Seiken Co Ltd) for nasopharyngeal swab fluid testing as the gold standard. Patients who presented weakly positive results in the rapid antigen test were excluded.

### Data Preparation

An automated medical interview system with an electronic template was introduced to standardize the entry of clinical symptoms, and the patient’s parents completed the form before the hospital visit. A group of highly trained general pediatric attending physicians with over 15 years of clinical experience created the template for entering symptoms and signs, allowing parents to select up to 3 symptoms per entry. Once a symptom was selected, the system presented additional questions based on the selection, with all responses except temperature being optional. Therefore, the status of each symptom, along with the number of elapsed days since symptom onset, was recorded as a categorical variable. Additional questions for each symptom were presented differently for each age group; all questions and options used are listed in Multimedia Appendix 1. The feature set was solely based on information from the electronic template and did not include any additional data from the examination or treatment. Based on medical insights, the symptoms introduced to the models were limited to cough, runny nose or nasal congestion, and wheezing, using only baseline characteristics and overall health status features. Statistical feature selection was not performed.

### Experimental Process

We developed and evaluated a machine learning model that outputs binary information on RSV infection based on symptom and sign information. Users can obtain information about RSV infection status by entering symptoms to determine whether to seek medical attention.

### Models

Random forest, extreme gradient boosting (XGBoost), and support vector machine models were used to determine appropriate machine learning algorithms. We used grid search in a hyperparameter space for all classifiers, optimizing the hyperparameters based on 10-fold cross-validation. The area under the receiver operating characteristic curve (AUC-ROC) was calculated for each model using the optimized hyperparameters. Finally, we selected the machine learning algorithm and its corresponding hyperparameters that performed best.

### Model Performance Evaluation

Model performance was assessed through calibration and discrimination. Calibration was evaluated graphically using a calibration plot and Hosmer-Lemeshow test with 10 groups, where *P*<.05 indicated a poor model fit. The AUC-ROC, sensitivity, and specificity were used to evaluate model discrimination ability. Sensitivity and specificity were calculated using the Youden index, and performance was calculated under conditions, where 1 parameter was fixed to be equivalent to that of the rapid antigen test. Additionally, we calculated model discriminatory power based on the number of elapsed days since the onset of illness to assess the use of rapid patient isolation. The performance of a valid model should not be considerably worse even in short periods after disease onset. The discrimination metrics of the final model were evaluated to estimate stability using the 1000 times bootstrap method. This resampling technique, which involves generating multiple bootstrap samples and using them to train and test the model, offers a robust estimate of evaluation indices even with small samples by correcting for optimism in the model’s performance [30].

To further assess the effect of additional symptom information on detection accuracy, we constructed a baseline model that classified patients based solely on the presence or absence of symptoms. As RSV infection is seasonal and its prevalence varies by season, a model excluding only the month of hospital visit was also created for comparison.

### Interpretability Evaluation

We evaluated the interpretability of the final model using Shapley additive explanations (SHAP), calculated using an algorithm that mimics the Shapley value used in game theory to evaluate the relative importance of each feature on discrimination performance while considering interactions. Therefore, it was used to corroborate the presence of essential variables and interactions. R (version 4.2.0; R Foundation for Statistical Computing) was used for all analyses.

## Results

### Patient Characteristics

**Figure 1:**
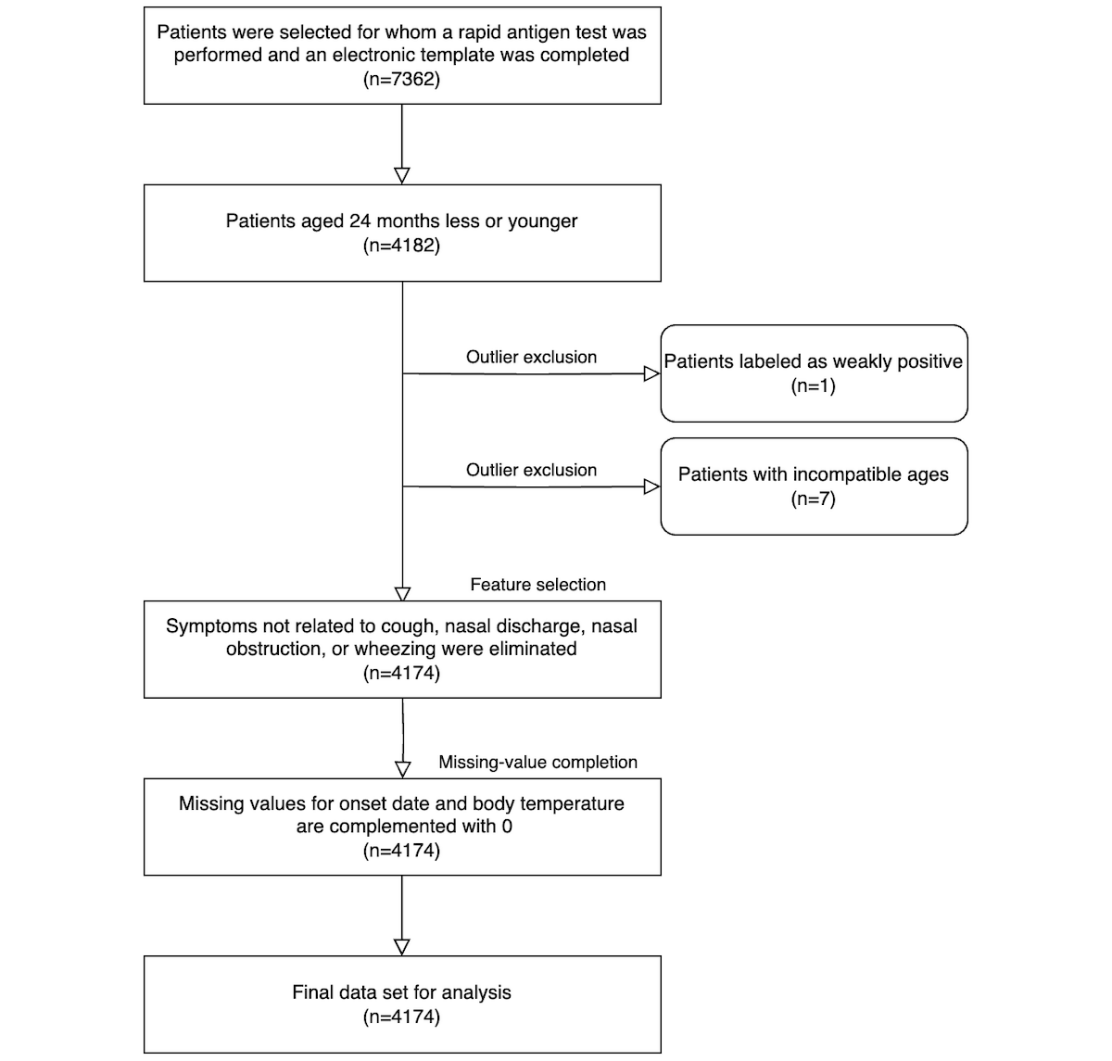
Sequence of steps in patient selection and data preprocessing.

Between January 2, 2009, and December 31, 2015, a total of 7362 patients underwent rapid antigen tests, and their parents provided the necessary information through an electronic template. Of these, 4182 patients who were aged 24 months or younger were included in the analysis. One patient with weak positive test results and 7 with inaccurate age information were excluded. Figure 1 depicts the process of patient exclusion, data selection, and missing value completion. Of the remaining 4174 patients, 619 (14.8%) were positive and 3555 (85.2%) were negative for RSV infection. Table 1 presents the demographic and clinical characteristics of patients. The details of the features used in the model are provided in Multimedia Appendix 1.

**Table 1:** Demographics and some important variables of the selected data (n=4174).

Characteristic	Values
Age (days), median (IQR)	340.0 (174.0-501.0)
Male, n (%)	2348 (56.3)
Respiratory syncytial virus-positive, n (%)	619 (14.8)
Current body temperature (°C) median (IQR)	38.0 (37.0-39.0)
Maximum body temperature (°C) median (IQR)	39.0 (38.0-40.0)
Cough, n (%)	2323 (55.7)
Wheezing, n (%)	1388 (33.3)
Nasal discharge or nasal distraction, n (%)	1944 (46.7)

^a^RSV: respiratory syncytial virus.

### Machine Learning Algorithm Selection

During the algorithm selection process, XGBoost demonstrated the highest AUC-ROC at 0.825 (95% CI 0.772-0.875), outperforming other algorithms such as support vector machine and random forest, which showed AUC-ROCs of 0.704 (95% CI 0.665-0.740) and 0.798 (95% CI 0.756-0.857), respectively. Therefore, we adopted XGBoost for the proposed and baseline models.

### Calibration and Discrimination Ability of the Differential Models

The model with the XGBoost algorithm fit well visually, with good calibration in the Hosmer-Lemeshow goodness-of-fit test (*P*=.27). For discrimination ability, the AUC-ROC of the estimated model calculated with 1000 times bootstrap was 0.811 (95% CI 0.784-0.833). The sensitivity and specificity were 73.5% (95% CI 66.8%-79.4%) and 73.9% (95% CI 70.3%-77.2%), respectively. To validate the exclusion performance of the proposed model, the threshold was adjusted according to the discrimination performance of the rapid antigen test [28]. When the sensitivity was set to 71.6% (95% CI 64.8%-77.8%), approximately 68.7% (95% CI 65.4%-71.9%) of the total patients were predicted to be negative for RSV infection. Patient samples were predicted to be positive (6.9%, 95% CI 5.4%-8.5%) when the specificity was set to 96.6% (95% CI 95.3%-97.7%). For the baseline model, which was considered to determine the effect of additional symptom information on performance improvement, the AUC-ROC was 0.766 (95% CI 0.739-0.792) for the model that excluded the month of visit (model 2), 0.703 (95% CI 0.677-0.731) for the model that considered only the presence of symptoms (model 3), and 0.521 (95% CI 0.484-0.556) for the null model that relied solely on age (model 4). The discrimination performance of the proposed model is summarized in Table 2.

Model prediction performance is calculated by adjusting thresholds under different conditions. The scores are based on 1000 times bootstrap, with means and 95% CIs of each sample.

**Table 2:** Discrimination performance under different conditions.

Condition and metric		Values, mean (95% CI)
Overall (AUC-ROC^a^)		0.811 (0.784-0.833)
**Youden index maximized (with threshold: 0.142)**		
	Sensitivity	73.5 (68.8-79.4)
	Specificity	73.9 (70.3-77.2)
**Sensitivity equivalent to that of the rapid antigen test (with threshold: 0.152)**		
	Sensitivity	71.6 (64.8-77.8)
	Specificity	75.7 (72.0-78.9)
	Percentage predicted negative	68.7 (65.4-71.9)
**Specificity equivalent to that of the rapid antigen test (with threshold: 0.463)**		
	Sensitivity	27 (21.0-33.6)
	Specificity	96.6 (95.3-97.7)
	Percentage predicted positive	6.9 (5.4-8.5)

^a^AUC-ROC: area under the receiver operating characteristic curve.

### Discrimination Based on Days Since Onset

**Figure 2:**
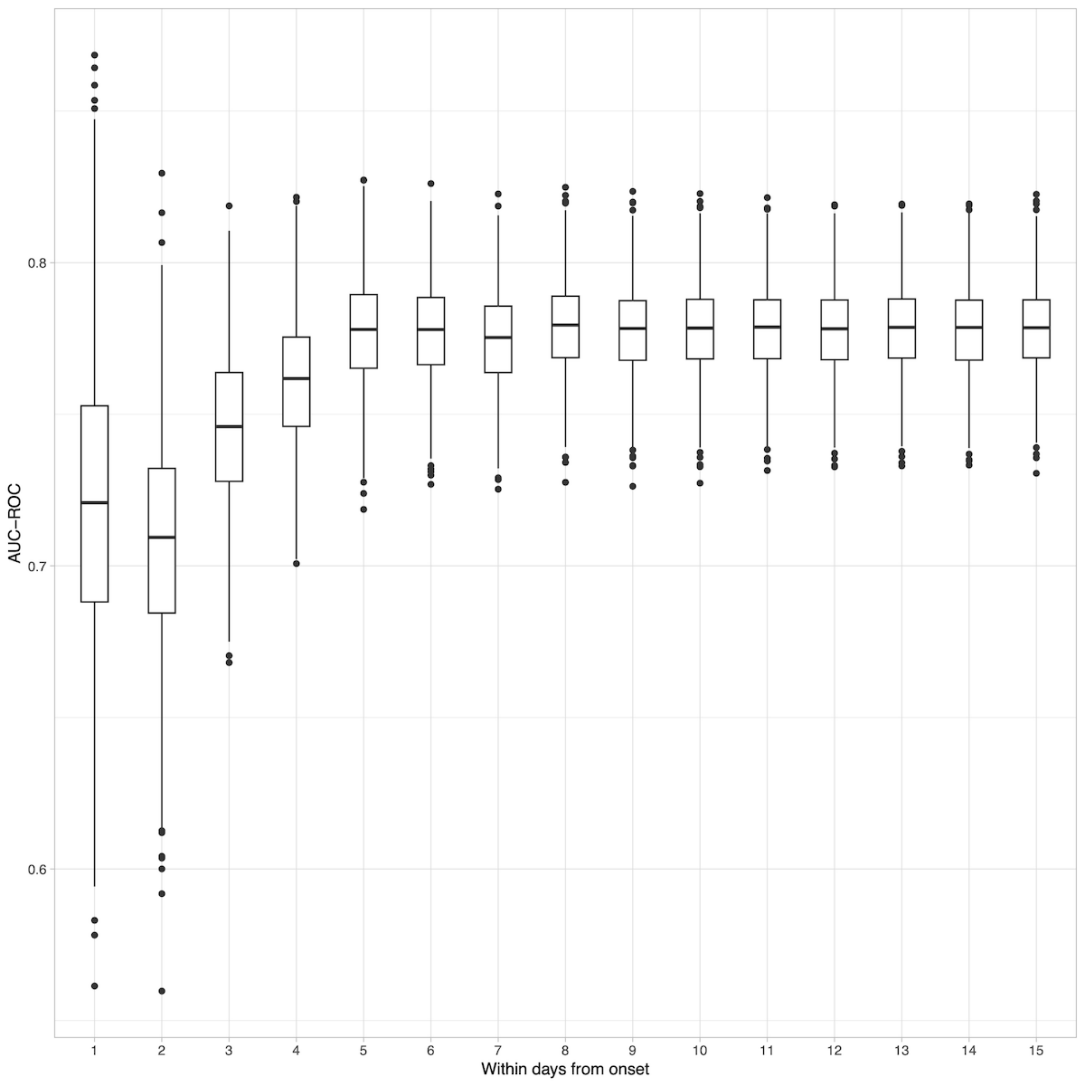
Area under the receiver operating characteristic curve (AUC-ROC) for elapsed days in the proposed model.

Elapsed time was measured by counting the number of days since the initial onset of symptoms. A total of 46.8% (n=1952) of patients reported that symptoms such as cough, nasal discharge, or wheezing began within 3 days, and 77.4% (n=3231) of RSV-positive patients developed these symptoms within 8 days. The AUC-ROC was 0.721 (95% CI 0.628-0.815) for patients on the day of symptom onset and 0.746 (95% CI 0.694-0.794) and 0.779 (95% CI 0.749-0.808) for patients within 3 and 8 days of onset, respectively. To assess the robustness of these AUC-ROC values, 1000 bootstrap samples were used for computation, as depicted in the box and whisker plot in Figure 2. This figure illustrates the distribution of AUC-ROC values across the elapsed days since symptom onset, highlighting that the variance in AUC-ROC values diminishes as the sample size increases with more elapsed days.

### Interpretability of the Final Model

**Figure 3:**
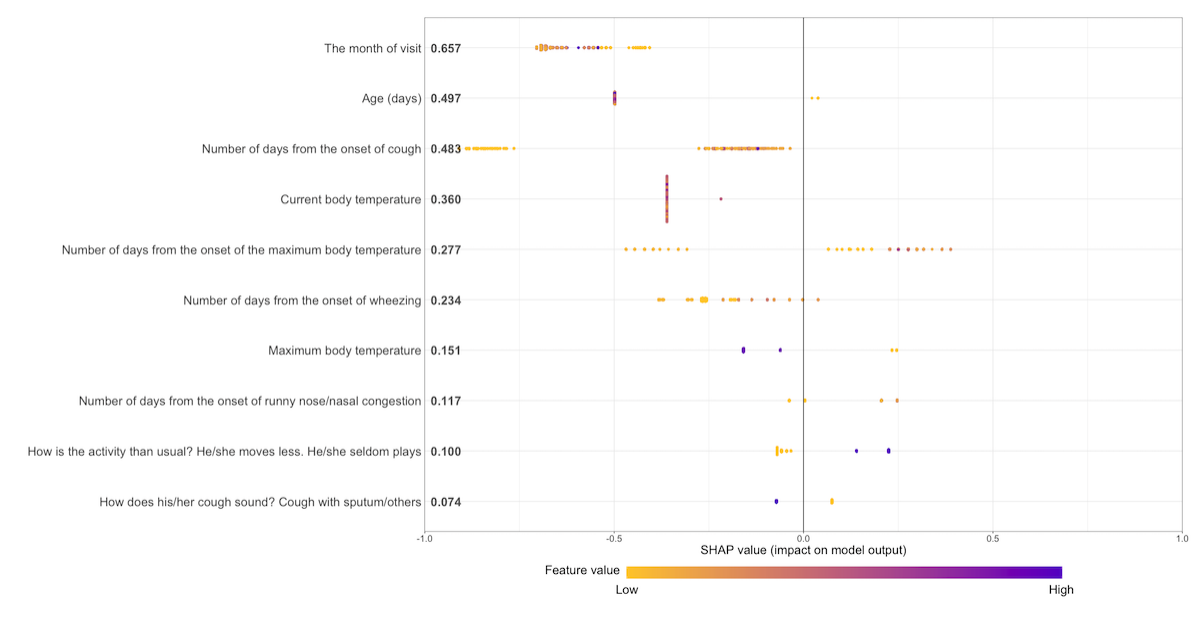
Average Shapley additive explanations (SHAP) value for each feature from the top in order of importance.

Variables contributing to the prediction were examined using SHAP. The variable that primarily contributed to the relative prediction performance was the month of visit, followed by the number of days from the onset of cough and maximum body temperature. Based on the Beeswarm plot, the number of days from the onset of the cough variable exhibited bifurcation along the x-axis, indicating variability in its impact on the model’s output. However, age or current body temperature did not show a clear trend between the feature value and SHAP (Figure 3).

## Discussion

### Principal Results

We developed a machine learning tool that leverages longitudinal diversity of symptoms for the remote detection of RSV infection at home. The model that incorporated the time course of the onset of symptoms and their evolution (model 2) exhibited enhanced discriminative performance. However, our final model, including information on onset days and month of visit (model 1), performed effectively in terms of sensitivity and specificity. By adjusting the threshold based on 2 exclusion criteria set at the same level as the rapid antigen test, this model applied thresholds of 0.152 or 0.463, thereby performing an exclusion accuracy comparable to the standard RSV detection method and successfully identifying 75.6% of infected patients for exclusion. This approach could potentially reduce the need for further confirmatory testing—most positive cases comprised samples with values above the threshold based on high sensitivity. Samples with values below the threshold had almost no positives and could be considered negatives. Samples with values below the threshold based on high specificity comprised mostly of negatives, indicating that samples with values above the threshold could be considered as positives. Nevertheless, rapid testing should be continued for patients who fall within the defined thresholds. The consistency between the estimated positive probability and the actual percentage was confirmed using calibration. Hence, there is no reason to perform additional testing on samples labeled based on the 2 thresholds.

When considering the number of days since disease onset, our proposed model demonstrated an AUC-ROC of approximately 0.721 for patients who began to experience symptoms on day 1, which is the visiting day, showing particularly high accuracy when including patients with symptoms that emerged within 5 days. The results indicate that discriminatory ability improved within approximately 6 days; however, it was high even on the day of onset.

We examined the characteristics essential for model identification. The month of visit was the most crucial variable; as RSV infection has a clear seasonality, the model reflected a tendency for RSV infection to occur more frequently in January. Although the peak period of RSV infection currently tends to be earlier, the presence of seasonality itself cannot be negated [31]. For the days of onset variable, there was a clear difference in the presence or absence of symptoms, but we could not identify a clear pattern in the number of days of symptom onset. This was also the case for age, suggesting that various interactions occurred among the characteristics. This observation suggests that the machine learning model ultimately captured additional symptom-related questions generated by skilled pediatricians and the complex onset patterns of patient underlying characteristics. This finding was also confirmed by the apparent difference in discrimination performance between a simple symptom-only model (model 3) and a model with additional symptom and patient information (model 2).

### Comparison With Previous Studies

The model used here showed a higher discriminative performance than those used in other studies on symptom-based RSV infection detection. For children, a model with 80% sensitivity, 68% specificity, and an AUC-ROC of 0.66 has been reported [15]. A model with 72.8% sensitivity and 73.2% specificity has also been reported; however, this model included x-ray and laboratory test results as features, which differed from the variables we used, where patients even outside the hospital could ascertain RSV infection by themselves [16]. Therefore, the results are considered noteworthy in terms of identification accuracy. To the best of our knowledge, this is the first study to include outpatients and obtain symptom information through nonmedical personnel, which is in contrast to previous studies that involved inpatients or data obtained by health care professionals [13-18].

Our results indicate that adequately accurate predictions can be acquired using machine learning and symptom information. The detection of RSV infection based solely on patient-reported symptoms is still in its early stages; however, our tool shows robust capabilities in distinguishing positive and negative results. The tool estimates RSV infection based on symptom data and progression entered by the patient’s caregivers at home, indicating that the intervention of medical personnel is not required. This remote detection strategy could potentially reduce the risk of nosocomial infections and physical burden on children. Moreover, by applying this tool, isolation measures can be implemented before visiting a medical facility. This will allow RSV infections to be detected at home, reducing the need to visit hospitals, thereby preventing secondary and nosocomial infections and reducing the burden on health care providers, especially during an epidemic, and protect them from coinfection with RSV and severe acute respiratory syndrome coronavirus 2 [32]. In addition, it may limit the spread of the virus in the community.

Another unique feature of this study is that the model was developed based on 3 primary symptoms: cough, nasal discharge, and wheezing, which were selected and extracted from the system based on the clinical manifestations of RSV infection. Therefore, although other symptoms were recorded in the electronic template, they were not used here based on clinical rationale.

### Limitations

There are several limitations to this study. First, participant demographics were limited as this study was conducted at a single institution. However, it is worth noting that Japan’s insurance system, which allows for free access, may mitigate potential economic-related biases in our findings. Additionally, the rapid antigen test may yield false positives when there is a low disease incidence [33,34]. Thus, the final diagnostic result, used as the gold standard here, may vary. The RSV prevalence pattern may already be changing and combining this with a surveillance system would further improve accuracy. Furthermore, model calibration must be confirmed based on race to assess performance differences between races when introduced to populations with widely varying demographics. In this study, we used a single cohort for internal validation and did not perform external validation, necessitating further testing on additional data sets to confirm generalizability.

### Future Research Directions

Future prospective studies are required to assess the generalizability of this algorithm to all patients because they may differ from the retrospective cohort used in this study. The model developed in this study is specific for RSV infection; however, similar methods may be used to construct models to detect infections with other viruses using their respective symptoms.

### Conclusions

Our detection tool was based on patient-reported symptoms and basic attribute information; nevertheless, it effectively detected RSV infection. Furthermore, our findings highlight the necessity to develop machine learning models and support the use of structural data for capturing complex patterns for symptom-based detection of RSV infection. The presented model leverages the distinct temporal patterns of RSV symptoms, allowing accurate identification of the infection even at early stages and with symptom evolution. Health care providers can perform model analysis before an outpatient visit to direct infected patients to home treatment or an appropriate isolation cohort. Applying this model to other settings can validate a standardized and comprehensive approach to improve RSV infection detection at home, and it could then be applied to other viruses.

## Appendix

Multimedia Appendix 1 All questions and options for model development.

## Abbreviations

AUC-ROC: area under the receiver operating characteristic curve
RSV: respiratory syncytial virus
SHAP: Shapley additive explanations
TRIPOD: Transparent Reporting of a Multivariable Prediction Model for Individual Prognosis or Diagnosis
XGBoost: extreme gradient boosting

## Data Availability

The data sets generated and analyzed during this study are available from the corresponding author on reasonable request.
