# Supplementary material for: Novel Approach for Detecting Respiratory Syncytial Virus in Pediatric Patients Using Machine Learning Models Based on Patient-Reported Symptoms: Model Development and Validation Study
Source: JMIR Form Res. 2024 Apr 12;8:e52412. doi: 10.2196/52412 (PMC11053391; doi:10.2196/52412)
Supplement: Multimedia Appendix 1 [file formative_v8i1e52412_app1.docx]

| **Baseline Characteristics / Symptoms / General Conditions** | **Age** | **Q** | **A** |
| --- | --- | --- | --- |
| Baseline Characteristics | All ages | Age (Days) |  |
|  |  | Sex |  |
|  |  | Body weight |  |
|  |  | The month of visit |  |
| Cough | 0 to 2 months of age | How is the severity of cough? | He/She vomits after coughing. |
|  |  |  | It is just a concern. |
|  |  |  | He/She is breathing fast and looks very painful. |
|  |  | How does his/her cough sound? | High pitch cough./Cough like barking dog. |
|  |  |  | Cough with sputum./Others |
|  |  |  | Normal cough/Mild cough. |
|  |  | How long does it last? | It is persistent once it starts. |
|  |  |  | Between 1 and 3. |
|  |  |  | It doesn't persist. |
|  | 3 to 4 months of age | How is the severity of cough? | He/She vomits after coughing. |
|  |  |  | It is just a concern. |
|  |  |  | He/She is breathing fast and looks very painful. |
|  |  | How does his/her cough sound? | High pitch cough./Cough like barking dog. |
|  |  |  | Cough with sputum./Others |
|  |  |  | Normal cough/Mild cough. |
|  |  | How long does it last? | It is persistent once it starts. |
|  |  |  | Between 1 and 3. |
|  |  |  | It doesn't persist. |
|  | 5 to 12 months of age | How is the severity of cough? | He/She vomits after coughing. |
|  |  |  | It is just a concern. |
|  |  |  | He/She is breathing fast and looks very painful. |
|  |  | How does his/her cough sound? | High pitch cough./Cough like barking dog. |
|  |  |  | Cough with sputum./Others |
|  |  |  | Normal cough/Mild cough. |
|  |  | How long does it last? | It is persistent once it starts. |
|  |  |  | Between 1 and 3. |
|  |  |  | It doesn't persist. |
|  | 13 months or older | Is there any trouble sleeping? | He/She can't sleep at all . |
|  |  |  | He/She wakes up several times a night. |
|  |  |  | He/She can sleep. |
|  |  | How does it sound when you put your ears on his/her chest? | I hear wheezing sound. |
|  |  |  | I don't hear much./I don't know. |
|  |  |  | I don't hear anything. (as usual) |
|  |  | How does his/her cough sound? | Hoarse high pitch cough. |
|  |  |  | Cough with sputum./Others |
|  |  |  | Normal cough/Mild cough. |
| Wheezing | 0 to 2 months of age | Can he/she sleep well? | He/She rarely sleeps. |
|  |  |  | He/She wakes up several times at night. |
|  |  |  | He/She can sleep. |
|  |  | When do you hear his/her wheezing? | It is heard when he/she breathes in or out. |
|  |  |  | It doesn't sound so loud. |
|  |  |  | I don't know. It doesn't sound always in the same way. |
|  |  | How long does it last? /How does it changed? | It started today./It is gettign worse. |
|  |  |  | For several days. |
|  |  |  | Fore more than 3 days |
|  | 3 to 4 months of age | Can he/she sleep well? | He/She can't sleep at all. |
|  |  |  | He/She wakes up several times at night. |
|  |  |  | He/She can sleep. |
|  |  | When do you hear his/her wheezing? | It is heard when he/she breathes in or out. |
|  |  |  | It doesn't sound so loud. |
|  |  |  | I don't know. It doesn't sound always in the same way. |
|  |  | How long does it last? /How does it changed? | It started today./It is gettign worse. |
|  |  |  | For several days. |
|  |  |  | Fore more than 3 days |
|  | 5 to 12 months of age | Can he/she sleep well? | He/She can't sleep at all. |
|  |  |  | He/She wakes up several times at night. |
|  |  |  | He/She can sleep. |
|  |  | When do you hear his/her wheezing? | It is heard when he/she breathes in or out. |
|  |  |  | I don't know. It doesn't sound always in the same way. |
|  |  |  | It doesn't sound so loud. |
|  |  | How long does it last? /How does it changed? | It started today./It is gettign worse. |
|  |  |  | For several days. |
|  |  |  | Fore more than 3 days |
|  | 13 months or older | Can he/she sleep well? | He/She rarely sleeps. |
|  |  |  | He/She wakes up several times at night. |
|  |  |  | He/She can sleep. |
|  |  | How is he/she breathing? | His/Her shoulder/belly moves everytime he/she breathes. |
|  |  |  | Between 1and 3. |
|  |  |  | He/She breathes normally. |
|  |  | How long does it last? /How does it changed? | It is gettign worse. |
|  |  |  | It hasn't cahnged. |
|  |  |  | It is getting better. |
| Runny nose/Nasal congestion | 0 to 2 months of age | Does he/she have nasal congestion? | Yes. His/Her sucking is disturbed because of nasal congestion. |
|  |  |  | Yes. He/She has some congestion but he/she can drink milk. |
|  |  |  | No. |
|  |  | How does his/her nasal discharge look? | He/She has some congestion but no discharge. |
|  |  |  | It has some color and sticky. |
|  |  |  | Water-Clear./Others. |
|  |  | Is there any associated symptoms? | He/She is breathing fast and his/her face looks pale. |
|  |  |  | He/She is breathing fast and his/her face looks normal. |
|  |  |  | He/She is breathing easy. |
|  | 3 to 12 months of age | When did it start? | 1 week or more than 1 week ago. |
|  |  |  | 3-6 days ago. |
|  |  |  | A few days ago. |
|  |  | Does he/she have nasal congestion? | Yes. His/Her drinking water is disturbed because of nasal congestion. |
|  |  |  | Yes. He/She has some congestion but he/she can drink water. |
|  |  |  | No. |
|  |  | How does his/her nasal discharge look? | He/She has some congestion but no discharge. |
|  |  |  | It has some color and sticky. |
|  |  |  | Water-Clear./Others. |
|  | 13 months or older | When did it start? | More than 2 weeks ago. |
|  |  |  | Several days ago. |
|  |  |  | Today. |
|  |  | Does he/she have nasal congestion? | Yes. His/Her sleep is disturbed because of nasal congestion. |
|  |  |  | Yes, he/she has some congestion. |
|  |  |  | No. |
|  |  | How does his/her nasal discharge look? | He/She has some congestion but no discharge. |
|  |  |  | It has some color and sticky. |
|  |  |  | Water-Clear./Others. |
| Relation to external factors | 0 to 24 months of age | Are the symptoms of this time are related to external factors such as injury or accident? | No. |
|  |  |  | Probably no. |
|  |  |  | Yes. |
| State of consciousness | 0 to 4 months of age | How is the state of consciousness than usual? | He/she made no response when I pinched his/her sole of foot as strongly as the trace of pinch did not disappear. |
|  |  |  | When I pinched, he/she responded with a groan. But, cries weakly. |
|  |  |  | When I pinched, he/she responded and cried hard / He/she remains unchanged. |
|  | 5 to 6 months of age | How is the state of consciousness than usual? | When I pinched, he/she responded. But, continued to sleep. |
|  |  |  | When I pinched, he/she responded. But, cried weakly. |
|  |  |  | When I pinched, he/she responded and cried hard / He/she remains almost unchanged. |
|  | 7 to 12 months of age | How is the state of consciousness than usual? | He/she made no response when I pinched his/her hand or foot as strongly as the trace of pinch did not disappear. |
|  |  |  | When I pinched, he/she responded. But, falls asleep immediately. |
|  |  |  | When I pinched, he/she cries hard / He/she remains almost unchanged. |
|  | 13 months or older | How is the state of consciousness than usual? | He/she made no response when I pinched his/her hand or foot as strongly as the trace of pinch did not disappear / When I pinched, he/she just moved without uttering a word. |
|  |  |  | When I pinched, he/she responded with a groan. But, continued to sleep. |
|  |  |  | When I pinched, he/she responded apparently / He/she remains almost unchanged. |
| Respiratory state | 0 to 4 months of age | How is the lip color and the respiratory state? | His/her Lips are bluish-purple / He/she stops breathing / His/her epigastrium balloons repeatedly. |
|  |  |  | His/her lips color are not bad, but nose twitches at each time of breathing / He/she grows with each breath. |
|  |  |  | He/she breathes easier when he/she takes his/her nasal discharge / He/she remains almost unchanged. |
|  | 5 to 6 months of age | How is the lip color and the respiratory state? | His/her Lips are bluish-purple / He/she stops breathing. |
|  |  |  | His/her nose twitches at each time of breathing / His/her epigastrium balloons repeatedly. |
|  |  |  | He/she breathes easier when he/she takes his/her nasal discharge / He/she remains almost unchanged. |
|  | 7 to 12 months of age | How is the respiratory state? | He/she stops breathing / He/she is not breathing. |
|  |  |  | His/her nose twitches at each time of breathing / His/her epigastrium balloons repeatedly. |
|  |  |  | He/she remains almost unchanged. |
|  | 13 months or older | How is the respiratory state? | He/she stops breathing / He/she is not breathing. |
|  |  |  | His/her nose twitches at each time of breathing / His/her epigastrium balloons repeatedly. |
|  |  |  | He/she remains almost unchanged. |
| State of activity | 0 to 12 months of age | How is the activity than usual? | He/she does not move at all, whatever I do for him/her. |
|  |  |  | He/she moves less. / He/she moves poor. |
|  |  |  | He/she moves well / He/she remains almost unchanged. |
|  | 13 months or older | How is the activity than usual? | He/she does not move at all, whatever I do for him/her. |
|  |  |  | He/she moves less. / He/she seldom plays. |
|  |  |  | He/she remains almost unchanged. |
| Status of intake | 0 to 6 months of age | How is breast milk or formula drinking/ sucking compared to usual? | He/she doesn’t drink at all / He/she doesn’t suck at all. |
|  |  |  | About half of his/her usual level/weak sucking |
|  |  |  | He/she can drink as much as usual / sucking is almost unchanged. |
|  | 7 to 12 months of age | Is he/she drinking enough water than usual? | He/she doesn’t drink and eat at all / He/she has not urinated for more than half a day. |
|  |  |  | He/she takes drinks and foods less than half of normal a day. |
|  |  |  | He/she remains almost unchanged. |
|  | 13 months or older | Is he/she drinking enough water than usual? | He/she doesn’t drink and eat at all / He/she has not urinated for more than half a day. |
|  |  |  | He/she takes drinks and foods less than half of normal a day. |
|  |  |  | He/she remains almost unchanged. |
| Body Temperature | All ages | Current body temperature |  |
|  |  | Maximum body temperature |  |
| Longitudinal characteristics of symptoms | All ages | Number of days from the onset of cough |  |
|  |  | Number of days from the onset of runny nose/nasal congestion |  |
|  |  | Number of days from the onset of wheezing |  |
|  |  | Number of days from the onset of the maximum body temperature |  |
